# “Feeling at home in Vanuatu”: Integration of newcomers from the East during the last millennium

**DOI:** 10.1371/journal.pone.0290465

**Published:** 2024-01-31

**Authors:** Wanda Zinger, Frédérique Valentin, Matthew Spriggs, Stuart Bedford, James L. Flexner, Edson Willie, Takaronga Kuautonga, Florent Détroit

**Affiliations:** 1 Archaeo- and Palaeogenetics Group, Institute for Archaeological Sciences, Tübingen University, Tübingen, Germany; 2 UMR 8068 TEMPS/CNRS/ Université Paris1 Panthéon Sorbonne/ Université Paris Nanterre/ Ministère de la Culture, MSH Mondes, Nanterre, France; 3 School of Archaeology and Anthropology, College of Arts and Social Sciences, The Australian National University, Canberra, ACT, Australia; 4 School of Culture, History & Language, College of Asia and the Pacific, The Australian National University, Canberra, ACT, Australia; 5 Max Planck Institute for Evolutionary Anthropology Department of Linguistic and Cultural Evolution, Leipzig, Germany; 6 Department of Archaeology, School of Philosophical and Historical Inquiry, University of Sydney, Sydney, Australia; 7 Vanuatu Cultural Centre Port Vila, Port Vila, Vanuatu; German Archaeological Institute: Deutsches Archaologisches Institut, GERMANY

## Abstract

Several localities across the Vanuatu archipelago (Melanesia), so-called ‘Polynesian Outliers’, are inhabited by communities that display Polynesian linguistic and cultural features although being located outside the Polynesian Triangle. Several introductions of Polynesian genetic components to Central and Southern Vanuatu during the last millenium have resulted in the cultural distinctiveness observed among the Polynesian Outliers in Vanuatu. However, social, political or economic process surrounding the exchange of genes between Polynesian and local individuals remain unidentified. Recent bioanthropological studies suggest the existence of female mobilities from neighboring regions to Vanuatu but also to the Polynesian Outliers of Taumako (Solomon Islands) within patrilocal societies. We aim to examine the hypothesis that Polynesian biological affinities observed in ancient individuals from Vanuatu are gendered or sex-specific, and that some of the Polynesian migrations during the last millennium may have involved practices of exogamy. By reconstructing phenotypes and biological identities from 13 archaeologically-recovered human skulls (400–300 years ago) from “Polynesian-related” regions of Vanuatu, we provide new insights to better contextualize the settlement patterns of Polynesian individuals. Eastern-Pacific associated phenotype are observable in 4 women from the Eretok burial complex (Efate region) and the Polynesian Outlier of Futuna, who were buried in close proximity to individuals with Western-Pacific associated phenotype. We suggest that close integration of individuals from the East into the local Vanuatu society, as well as the practice of exogamy, might have been key processes contributing to the preservation of Polynesian cultural features in Vanuatu over the past millennium. Our finding are cross-referenced with oral records from these two areas, as well as the known genetic makeup of the Vanuatu Polynesian Outliers.

## Introduction

**Fig 1:**
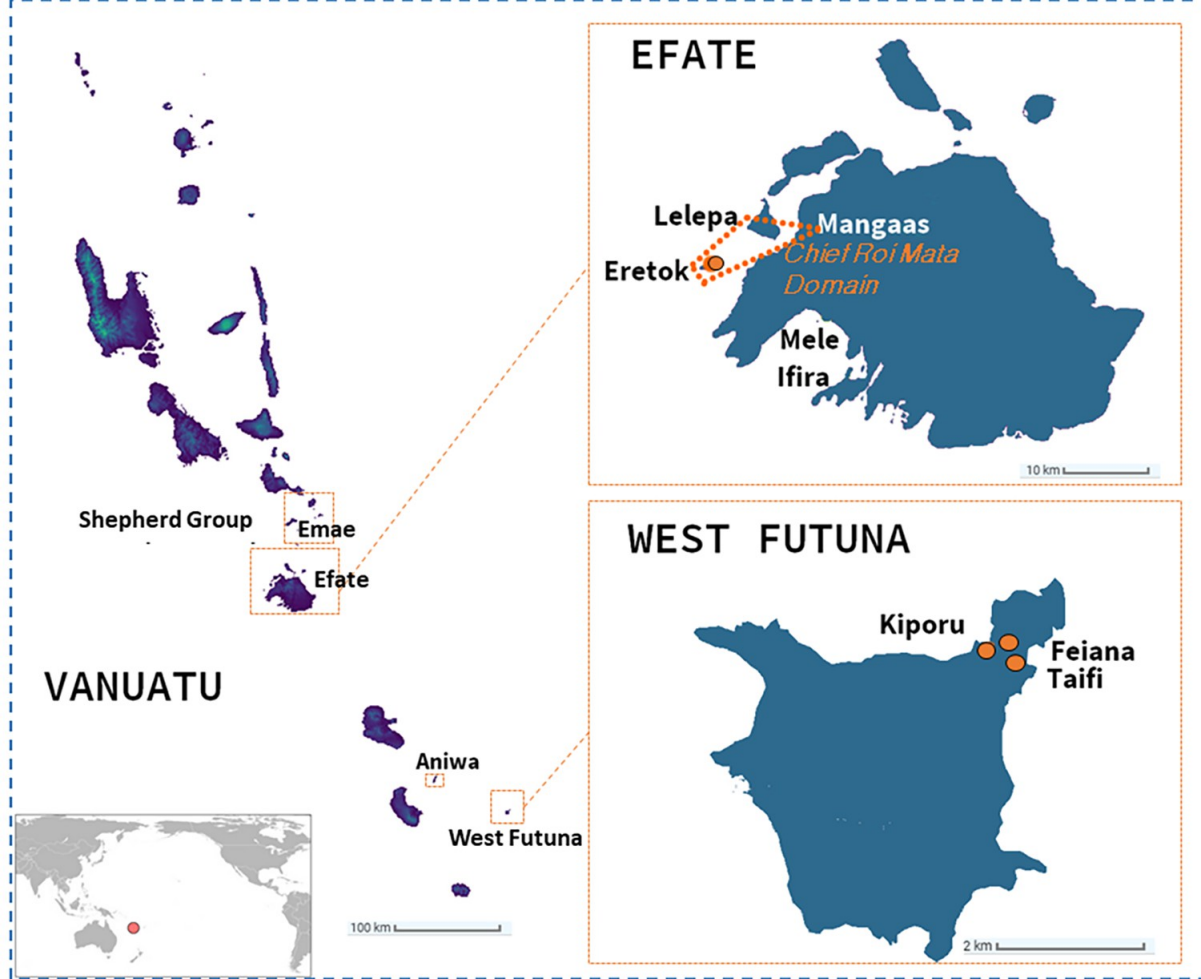
The Vanuatu archipelago with a focus on the sites of the CRMD and the Polynesian Outlier of West Futuna. Orange dashed squares correspond to regions which comprise communities with a Polynesian language. Orange dots correspond to the sites where the studied archaeological individuals have been recovered. Map was created from Shuttle Radar Topographic Mission coordinates available on CIAT-CSI SRTM website https://srtm.csi.cgiar.org [26].

The tremendous cultural diversity characterizing the Melanesian islands, including the Vanuatu archipelago, results from interactions between several populations of diverse origins [1–6]. Today, human societies living in five localities of the Vanuatu archipelago stand out from others because they speak a Polynesian language and their socio-cultural practices are related in part to Polynesian culture, even though they are located outside the Polynesian Triangle [7]. These localities, known as "Polynesian Outliers” [8] are found in Central Vanuatu on the small offshore island of Ifira, in Port Vila harbor (Efate), in the locality of Mele on the west coast of Efate, and on the island of Emae in the Shepherd group (Fig 1) [9–11]. In Southern Vanuatu, the Outlier communities are on the small islands of (West) Futuna and Aniwa [12–14]. Moreover, Polynesian influence spread over much larger areas of the archipelago beyond the localities where Polynesian languages are spoken [15, 16]. The degree of these influences varies depending on the locality they affect [17–20]. For example, in Southern Vanuatu, Polynesian loan-words associated with the consumption of kava (Piper methysticum), as well as references to Polynesian mythological heroes such as Maui (known as Mahjijiki or Mwatiktiki) and Tangaroa (Tangalua or Tagaro), demonstrate such influences [15, 21, 22]. In Central Vanuatu, Polynesian influences are evident in a highly structured chiefly system, a "feudal" type of landholding in the Shepherd group, and Polynesian vocabulary and place names [23]. For example, the origin of the chief title “roi”, associated with the UNESCO World Heritage Chief Roi Mata’s Domain (CRMD), is said to be Polynesian [24, 25].

The prevailing model of the origin and formation of the Polynesian Outliers suggests that individuals from Polynesia physically settled in these remote locations during the last millennium [8, 27, 28] see also [29]. The time-period of these dispersals was associated with the development of a distinctive phase of the Polynesian culture around 1000 BP in Western Polynesia [30]. The communities would have maintained a Polynesian language and cultural traits through generations thanks to the presence of individuals with Polynesian origins. For a long time, this model lacked solid evidence. Modern genetic analyses have shown that extant populations from the Polynesian Outliers are not biologically distinct from populations living in the surrounding islands of the Western Pacific, and they do not share more components with Polynesian populations than the populations of the other islands in the region not considered as Polynesian Outliers [3, 31–33]. Archaeological research on 12 Polynesian Outliers among the about twenty across Micronesia and Melanesia mentioned in the literature [8] has also not usually revealed archaeological layers containing material typically affiliated with Polynesian culture, although some Polynesian-associated objects such as basalt tools, ornaments, trolling lures and sea mammal teeth pendants have been identified [12, 34–40]. This situation is unique because linguistic and social features displayed by Polynesian Outlier communities in Vanuatu do not reflect their biological features or material culture. It is still unclear how and why Polynesian migrations triggered language shifts in some localities and not in others.

This paradoxical situation has motivated research into the origins of the Vanuatu Polynesian Outliers, yielding new results. Paleogenetic analysis by Lipson et al. (2020) [2] showed that 4 of 6 individuals dated to the last millennium CE from Efate (Chief Roi Mata Domain, CRMD) exhibit both local and Polynesian-derived ancestries, with different levels of admixture; with a time-period of introduction of "Polynesian-related" genetic components identified as occurring between 700–300 BP. On the other hand, Arauna et al. (2022) [41], based on the modern genome of 1433 contemporary Ni-Vanuatu individuals, suggests an earlier admixture date of between 1000 and 600 BP for the Polynesian Outliers of Vanuatu. These findings validate the introduction of Polynesian genetic components to Central Vanuatu during the last millennium. However, the particular social, political and economic circumstances surrounding the exchange of genes between Polynesian and local individuals remain unknown. A more complete description of the biological features and social identities of the individuals from Polynesian Outliers could contribute to a better understanding of the biological diversity in the region at that time and how people were connected to each other [42, 43]. The question of whether Polynesian components were incorporated through the physical establishment of Polynesian individuals in the region either within or at the margins of indigenous societies or were indirectly assimilated through social, commercial, or political exchanges with Polynesian regions remains a topic of ongoing discussion [44].

Recently, isotopic analysis of strontium contained in the teeth of 57 individuals from the Namu cemetery on Taumako Island, a Polynesian Outlier in the Solomon Islands, suggests female mobility from neighboring regions to Taumako within patrilocal societies [45]. Interestingly, one woman is not from the Solomon Islands, although her origin remains unknown. Matrimonial exchanges are good examples of a mechanism that allows newcomers to be integrated within a hosting community. The songs originating from the Polynesian Outliers of Futuna (Vanuatu), highlight the arrivals of foreign women on the island, either from the neighboring islands of Southern Vanuatu or more distant locations, and their inclusion within the local society through marriages [18, 21]. Arauna et al.’s 2022 [41] study confirms the present-day existence of marriages between individuals from geographically distant islands of Vanuatu, but in very small proportions (5.70% of the 287 analyzed couples), and endogamy does not seem to be the norm, although the individuals still share a high level of genetic relatedness.

In this paper, we propose to test the hypothesis that Polynesian biological affinities observed in ancient individuals from Vanuatu are gendered or sex-specific, and that some of the Polynesian migrations during the last millennium may have involved practices of exogamy (such as inter-regional marriages). Were Polynesian individuals involved in inter-regional matrimonial exchanges between Polynesia and Vanuatu? Was the practice of exogamy one of the processes that contributed to the formation of Polynesian Outliers in Vanuatu? What were the relationships between Melanesian and Polynesian communities? To gain insights into these issues, we undertook an analysis of the phenotype, described with morphometric techniques, of 13 individuals uncovered in archaeological sites related to the second millennium CE from two Polynesian-influenced regions of Vanuatu: CRMD (Eretok, Efate, Cental Vanuatu) and Futuna Island (South Vanuatu), compared to 232 individuals from regional reference series. We discuss our results taking into account their funerary context and their sex affiliation to assess whether Polynesian morphological orientations are sex-specific.

## Materials

**Fig 2:**
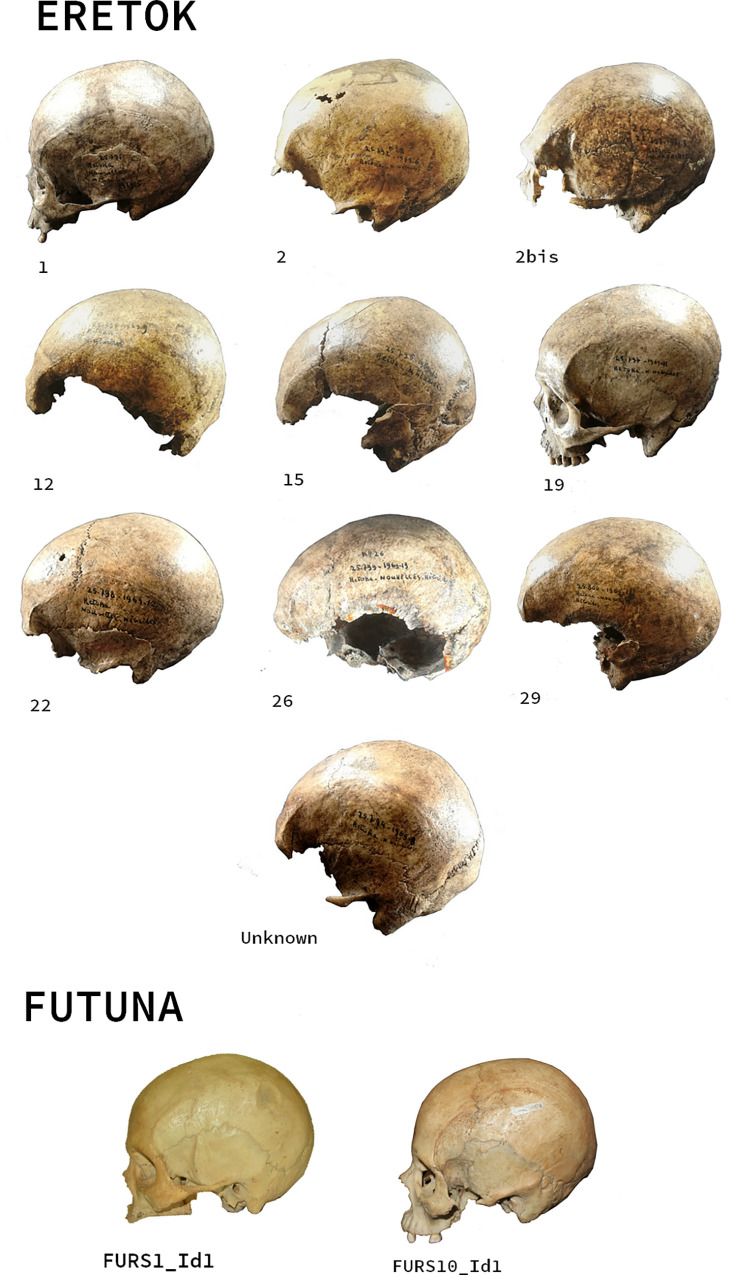
Examples of the archaeological skulls used in this study. The context of one individual from Eretok has been lost, it is identified by the expression: « Unknown ».

We have examined skeletal remains related to the second millennium CE uncovered in archaeological sites from two Polynesian-influenced regions of Vanuatu. The first is the burial complex of Roi Mata (Eretok) classified as a UNESCO World Heritage Site [46] for the concordance between oral traditions and remarkable archaeological findings [9, 23, 24]. At least 35 human burials richly decorated, dated to c. 400 cal BP [47], were interred simultaneously around the grave of the famous Chief Roi Mata, with the majority of individuals, certainly linked by matrimonial ties, buried in couples. It is considered as a classic case study of the practice of morts d’accompagnement [48], where people have voluntarily decided to accompany the death of a leader (according to oral records) [9]. The second is the Polynesian Outlier of Futuna, where burials related to the last millennium CE (c. 300–200 cal BP) were found on the floor of ten rock shelters. These represent changes in mortuary practices on the island over time [49], with earlier burials (c. 1100 cal BP) interred in the fill of the rock shelters. Our present analysis relies on ten adult skulls from the Roi Mata burial complex on Eretok, and three adult skulls derived from surface burials located in three rockshelters on Futuna, previously unpublished (Fig 2 and Table 1).

**Table 1:** Archaeological individuals analyzed in the study. The Eretok sample is housed in the Biological Anthropology collection of the Muséum National d’Histoire Naturelle (MNHN) (Paris). Burial number for Eretok sample were given by José Garanger [9].Sex was osteologically estimated [50], F = Female; M = Male; Und. = Undetermined. Two individuals from Futuna, collected by Mary Elizabeth and Richard Shutler [13], were studied at the Vanuatu Cultural Centre (VKS) (Vanuatu). The third (FURS13_Id1) was studied in situ, in rockshelter FURS13.

Region	Site/Date	N°burial	N° Individual (MNHN)	Sex estimation	N° Individual [3]
Efate, Central Vanuatu	Eretok	1	25791	F	l10969
		2	25792	F	
		2bis	25793	M	l10968
		12	25795	F	
		15	25796	F	
		19	25797	M	l14493
		22	25798	F	
		26	25799	Und.	
		29	25800	Probable female	
		Unknown	25794	F	
Futuna, South Vanuatu	Feiana	FURS1_Id1	-	F	
	Kiporu	FURS13_Id1	-	F	
	Taifi	FURS10_Id1	-	F	

We have co-analysed their morphometrical data with a comparative data set of 232 modern adult skulls from 11 populations distributed across the Pacific region. 120 modern skulls from Western-Pacific and 112 from Eastern-Pacific areas were used to represent the phenotypic variations of populations from Australia/Melanesia and populations from Polynesia (S1 Appendix and S1 Table). Genetic and morphological differences between insular populations from Melanesia and Polynesia have already been demonstrated and are mainly driven by the level of First Remote Oceanian (FRO) ancestry (i.e. most ancient individuals from Vanuatu and Tonga) among pacific islanders. Portion of FRO ancestry is higher in populations located in the eastern Pacific while western Pacific populations tend to exhibit a lower portion of FRO ancestry [1].The Polynesian sample includes populations from the eastern part of the Polynesian triangle in order to identify morphological features associated with culturally Polynesian populations and to optimize morphological differences between the Western Pacific and Eastern Pacific. We made this choice because our purpose is not to identify a source for the Polynesian migrations of the last millennium to the western Pacific but to detect Polynesian phenotypic variations in ancient individuals from Vanuatu. We report our findings using geographical terms in order to avoid terms which could imply that culture or language corresponds with biology in a one-to-one fashion. The analysis below is based on the idea that biological variation can be measured independently of language or culture, but that linguistic and cultural dynamics might then be interpreted from apparent trends in phenotypic difference. For this paper we thus decided to use two geographic-based expressions. “Eastern-Pacific” refers to the range of phenotypic variation that is typical of modern populations living in the part of Oceania further east than Fiji, Tonga and Samoa, the region considered as “Eastern-Polynesia”. “Western-Pacific” corresponds to modern populations living in the west part of Oceania that includes the regions of Australia and Island Melanesia.

## Methods

Cranial measurements can constitute useful biomarkers to understand population histories through morphological affinities, revealing affiliations among individuals inside a defined geographical and chronological area [51–54]. Morphometrics consist in recording the form of any element based on the equation “shape + size = form” [55]. Our comparative data set includes measurements taken on skulls of males and females (over 25 years old) in order to register the full diversity among the groups. Immature and young individuals have been excluded by the observation of the fusion-stage of the spheno-occipital synchondrosis [56]. Sex has been estimated on archaeological skulls with a method using visual assessment of cranial features [50].

A selection of 12 cranial linear measurements which are consistent with an optimal description of the form (size and shape) of the skulls and with the state of preservation of the archaeological specimens (measurements on frequently missing anatomical parts were discarded), were taken with a sliding calliper by one of us (WZ) (S2 Table). Instead of raw measurements we have used size-corrected measurements using Log Shape Ratios (LSRs) transformation [57] to highlight shape variations and reduce size effects in the analyses including that induced by sexual dimorphisme [58]. Size is poorly informative for our purpose, generally seen as being extremely influenced by sexual dimorphism [59, 60]. Shape variations are more subtle but much more informative regarding inherited morphological features and biological relatedness [61, 62].

We first analysed the dataset corresponding to the selection of 12 measurements (LSRs) for the whole sample of modern populations with Principal Component Analysis (PCA) and Between Group PCA (bgPCA) (calculated from the individual PC score) to assess its ability to give insightful information on population differences and affinities. Primary statistic for each population are available in S3 Table. In a second step, we analysed the 12 populations merged into two geographical groups: Eastern-Pacific (n = 113) and Western-Pacific (n = 121) which have been shown to encompass most of the variation within the region [52].

Archaeological individuals were projected as supplementary in the PCA and bgPCA analyses to observe their position in the shape space defined by the modern regional pool. Their measurements are available in S4 Table. Biological affinities of the archaeological specimens were assessed with Linear Discriminant Analyses (LDA) calculated with the individual PC scores of the PCA, with geographical origins as the discriminant factors (two groups) and leave-one-out cross validations [63, 64]. Statistical analyses and graphics were computed with R [65] using several functions adapted from [64] and the libraries ggplot2 [66] and FactoMIneR [67].

## Results

**Fig 3:**
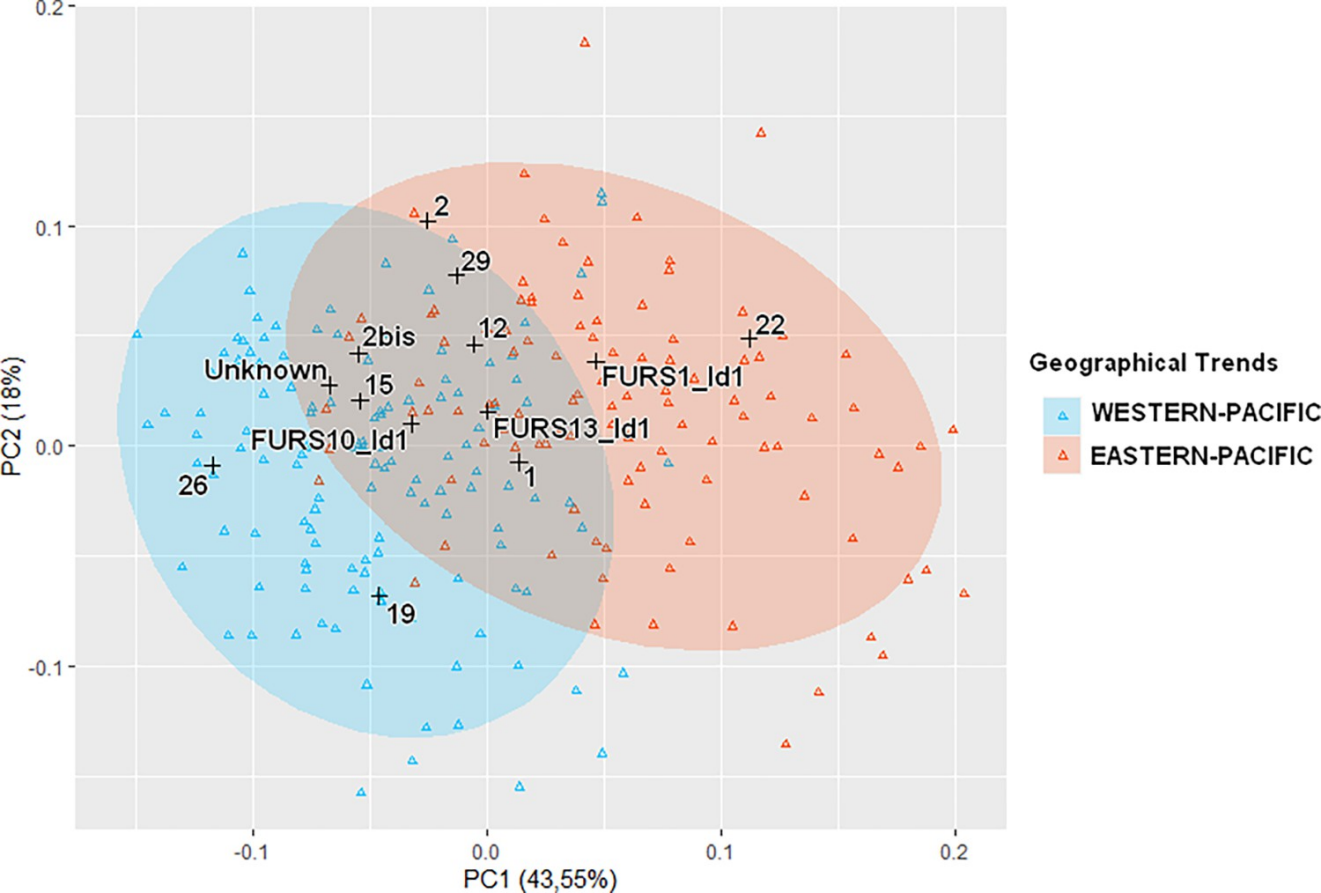
PC1-PC2 of 232 modern individuals from 11 populations merged into 2 geographical trends (Eastern and Western Pacific) with 13 archaeological individuals projected as supplementary (size-corrected measurements using *LSRs*, 61.55% of total variance, 95% ellipses).

Analysis of skull morphology and morphometrics of modern populations from Oceania in most studies shows a contrasting division into two groups, with a first group comprising populations from Island Melanesia and Australia and a second group including populations from Polynesia [52, 68]. Our BGPCA and PCA of 232 individuals from 11 populations (Papua New Guinea; Australia; Solomon Islands; Vanuatu; New Caledonia; Loyalty Islands; Wallis; Tuamotus; Tahiti; Marquesas and Easter Island), tend to confirm this geographical pattern of variation although showing a continuum from Western-Pacific populations to Eastern-Pacific populations (Fig 3 and S2 Appendix). There is an area of overlap indicating the existence of individuals with similar morphologies in both regions. As this analysis is intra-specific, overlap joining Western and Eastern populations could result from migrations and admixtures between individuals sharing a common history. However, outside of the area of overlap, phenotypic variations between Western and Eastern-Pacific populations are distinctive enough to reflect particular morphological differences between the geographical areas. These characteristics are used to determine whether the archaeological individuals from Eretok and West Futuna feature morphologies associated exclusively with Eastern or Western trends.

The 13 archaeological individuals (Table 1), projected as supplementary on the PCA plot (Fig 3), are all included in the range of Oceanian variation. Three (22; 2 and FURS1_Id1) display long and high skulls (short length of the parietal bone and a long length of the porion-bregma chord) like the modern individuals living in Eastern-Pacific islands. Those (19; 26) plotting with modern skulls from Australia and Melanesia, show low skulls and relatively narrow faces (S3 Appendix).

The archaeological individuals are largely distributed along the 2 PCs, suggesting heterogeneity among the sample. Two individuals clearly plot within the Western Pacific variation (19; 26) along the positive values of PC1. Three others plot within the Eastern Pacific variation (22; 2 and FURS1_Id1) close to the null values of both axes. The eight remaining individuals (1; 2bis; 12; 15; 29; Unknown; FURS10_Id1 and FURS13_Id1) plot in the ‘purple’ area where individuals of the two regions overlap on the negative values of PC1.

To compensate for the limited discriminant resolution of PCA, we used Linear Discriminant Analysis (LDA). Table 2 provides the percent of correct assignations of the modern skulls to their geographical origin given by the LDA. 87.5% of them are correctly assigned to their respective original region. This high percentage of correct assignations implies that the discriminant function computed with the 12 cranial measurements for 232 modern individuals divided into two geographical regions provides a good discrimination. This function was therefore used to evaluate the affinities of the archaeological individuals.

**Table 2:** Discriminant analysis: Assignations of modern individuals to two geographical groups (discriminant factor) after leave-one-out cross validation from the LDA of 232 modern skulls from Oceania.

Geographical group	Western Pacific	Eastern Pacific	Total	% correct
Western-Pacific	111	15	126	88%
Eastern-Pacific	14	92	106	86.7%
Total	125	107	232	87.5%

**Fig 4:**
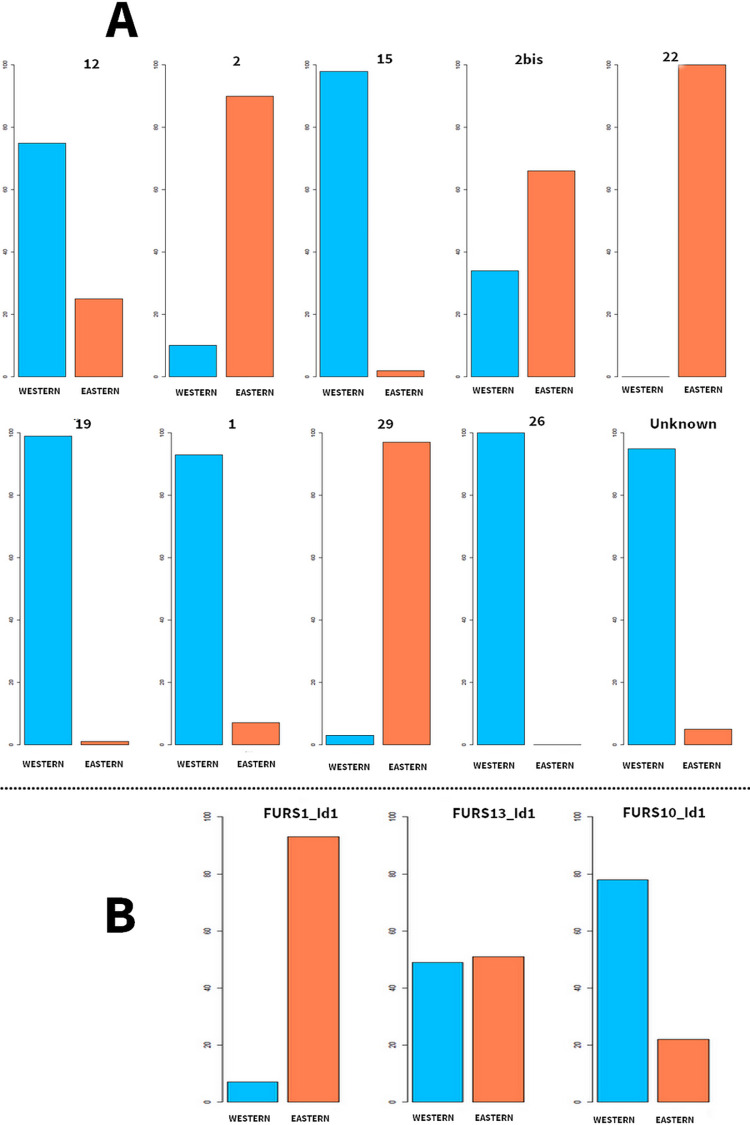
Morphological affinities of the archaeological individuals from Eretok (A); and Futuna (B): bar plot of the predicted probabilities of posterior assignations to the 2 geographical areas: Eastern-Pacific (Orange) and Western-Pacific (Blue).

Predicted classifications for the 13 archaeological individuals are presented in Fig 4 and S5 Table. One individual (FURS1_Id1) from Futuna displays 93% of Eastern-Pacific morphological affinities whereas the two others (FURS13_Id1 and FURS10_Id1) combine affinities with Western-Pacific and Eastern-Pacific populations (Table 1). Individuals from Eretok show variable profiles. Three display dominant morphological affinities with Eastern-Pacific variations: 2 (90%), 22 (100%) and 29 (97%); five with Western-Pacific variations: 1 (93%), 15 (98%), 19 (99%), 26 (100%) and Unknown (95%); and two (2bis and 12) display a combination of features associated with both regions. Three of these individuals (1, 2bis, 19) have ancient DNA profiles [3] which display some parallels with their respective morphological profiles. All three appear genetically composite with a FRO ancestry ranging coarsely between 20 and 40% (estimates from qpAdm).

Sex estimation [50] was coupled with biological affiliation to offer a more detailed profile for each archaeological individual. Interestingly, the four morphologically Eastern-affiliated individuals are females or a probable female (2, 22, 29 and FURS1_Id1) (Table 1).

## Discussion

Our results provide a biological picture of several individuals who lived c. 400 BP and c. 300 BP in Central and Southern Vanuatu. They highlight a large phenotypic diversity within each region. The nature of the trends we have identified provides a basis for discussing the modalities of ancient Polynesian arrivals and settlements in Vanuatu.

### The presence of the Polynesian phenotype in Vanuatu

Five individuals of Eretok (1, 12, 15, 19, 26, Unknown) and one individual from Futuna (FURS10_Id1) display a phenotype associated with modern populations from Australia and Island-Melanesia (Fig 4). Australo-Melanesian morphological features are documented in Northern, Central and Southern Vanuatu from soon after the earliest stages of human colonisation of the archipelago, at sites such as Marseille (Tanna) dated to 2650 BP, at Uripiv and Vao (Malakula) where burials date back to approximately 2500–2000 BP and at the site of Taplins (Efate) where burials dated to about 2300 BP [5, 69, 70]. These features are also documented in more recent periods in Central Vanuatu at the site of Mangaliliu (c. 600BP) and in present-day populations of Vanuatu. Based on these findings, we conclude that the 5 individuals displaying this phenotype at c. 400 and 300 BP are strongly affiliated with the phenotype. associated with populations present in Central and Northern Vanuatu from 2300 and 2500 BP respectively, though later arrivals from Northern Melanesia cannot be excluded.

In contrast, four other individuals of our sample exhibit a phenotype that is notably different from the ones generally observed in Vanuatu during both ancient and recent periods. Three of these individuals are from Eretok (29; 2 and 22) and one is from Futuna (FURS1_Id1). These individuals present a probability of assignation to Eastern-Pacific populations that range from 90% to 100%. Based on these high percentages, we reject the idea that they are affiliated with the earlier population of Vanuatu. Instead, our results suggest a strong affiliation to human groups living on Polynesian islands today. Our results therefore constitute direct evidence of the physical existence of individuals displaying a phenotype which can be considered as “Polynesian” in Central and Southern Vanuatu at c. 400 and c. 300 BP respectively.

Results of studies of skeletal remains from two other Outliers in Micronesia and Solomon Islands are inconclusive about the actual presence of a Polynesian phenotype among their populations. Houghton has described the presence of rocker jaws and flattening of the femoral shafts on six individuals from the Putau site (300–100 BP) on the Outlier of Kapingamarangi (Micronesia), which he thinks are specific to Polynesian populations [71]. However, Pietrusewsky [72] (:344) questions this view: “We do not know if such statements are accurate because […] detailed systematic studies of non-Polynesian skeletal populations are extremely rare”. Similarly, the results of studies of the Namu cemetery, on the Outlier of Taumako (Solomon Islands, 775–205 BP [35]) are ambiguous. Although Katayama [73] and Hougthon [74] detected what they consider to be Polynesian morphological traits in non-metric infracranial features and cranial and dental metric features. Pietrusewsky [75] contended, based on a sample of 18 crania (out of 77), that the morphology of these individuals is similar to that of modern Melanesian groups from Fiji, the Loyalty Islands and Santa Cruz.

### Antiquity of the Polynesian presence in Central and Southern Vanuatu

The presence in our sample of four individuals displaying a morphological profile that associates both Western and Eastern affinities (2bis, 12, FURS13_ID1 and FURS10_ID1) raises questions about the antiquity of the arrivals of migrants from the East. On the one hand, these individuals can be considered as displaying a phenotype that belongs to the general human morphological range of variations in Oceania without enough discriminant morphological features. On the other hand, they may have been the product of admixture between Western and Eastern individuals.

In this second view, the existence of individuals presenting a combination of features associated with both regions at Eretok suggests that Polynesian arrivals occurred before the time of Chief Roi Mata’s burial ceremony (c. 400 BP). In our study, individual 2bis displays 34% of morphological assignations related to Western-Pacific populations and 66% to Eastern populations. This result parallels the palaeogenetic result [2], indicating that the same individual (I10969 or 2bis) and other individuals from CRMD are modeled as having an excess of ancestry related to First Remote Oceanian (FRO) compared to other Efate individuals dated to the last 500 years. The average dates of admixture for these individuals is estimated at 20–30 generations earlier (i.e., ~1400–700 BP). This temporal range is consistent with the expected time of the first human dispersals from Polynesia into Southern Melanesia, as suggested for other Outliers like Tikopia [76, 77] or Taumako [35] based on archaeological evidence. This observation is interpreted [2] as the outcome of an early Polynesian genetic input. This further suggests in our case that the profile of individuals 2bis and 12 of Eretok, as well as of individuals FURS10_ID1 and FURS13_Id1 of West Futuna, are likely the result of admixture between Polynesian migrants and “Western-Pacific-like” individuals that had occurred well before c. 400 BP in Efate and c. 300 BP in West Futuna.

### Integration of Polynesian individuals in local Vanuatu social systems

**Fig 5:**
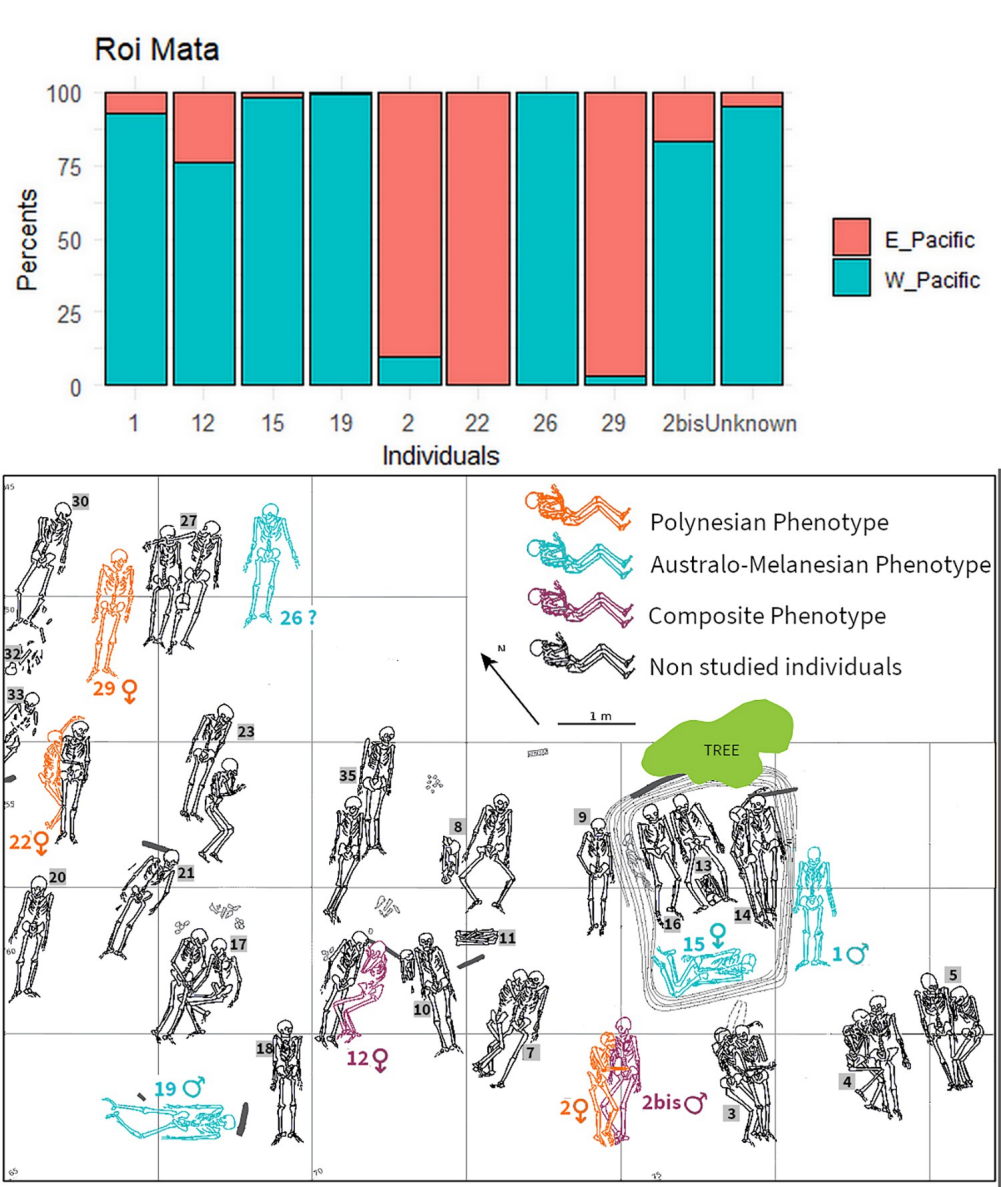
Map of the Chief Roi Mata burial complex (Eretok), after Garanger 1972: Spatial distribution of phenotypes and sex for 9 studied individuals. Archaeological context of the tenth individual is “Unknown”.

At Eretok, individuals presenting diverse morphological profiles, ranging from Eastern-Pacific affiliation to Western-Pacific affiliation, were all buried at the same time, with no discernible pattern according to their morphological affiliation. The Eastern affiliated individuals were not interred in a specific location within the site. They are distributed across the site among individuals showing various levels of Western-Pacific affinities (Fig 5). Body positions, associated artefacts and mortuary practices devoted to these individuals do not suggest that they received different treatment at death [9]. Chief Roi Mata is one of the most significant figures in the recent history of Vanuatu [20, 23]. He is acknowledged as the individual who brought about a peaceful environment to Efate and Shepherds islands after a prolonged era of strife. As per the oral traditions, Chief Roi Mata fell sick during a feast where people competed against each other, and because of his elevated status, he was interred on the secluded Eretok island along with his followers [23]. Organizing their funeral would have required a significant amount of time and the participation of communities from various parts of Central Vanuatu, who were willing to sacrifice several high-ranking people as part of the burial ceremony [20]. Analysis of the material associated with the three individuals (22, 29 and 2) displaying Eastern-Pacific morphological variations, suggest they would be representative of tributary chiefs or functionaries status, with the presence of worked stones, armband (burial 22 and 2) and 34 trochus shell-bracelets (burial 29), all considered as marks of rank [9, 23]. Whether the leader buried at Eretok was the peace-making Roi Mata of oral tradition or another chief of the same lineage is an open question [24]. Nevertheless, the fact that Eastern-Pacific affiliated individuals were included in an important funerary ceremony involving “morts d’accompagnement” [48] indicates that they held significant social and political status within the community and were not excluded despite their different physical appearances and origins.

In Futuna, the rockshelter in which the Eastern-Pacific affiliated individual is buried (FURS 1, Feiana) contained human remains belonging to at least three other adults and two children. As in Eretok, she was not the subject of a different mortuary treatment, being deposited on the surface of the floor of the rockshelter exactly like the other individuals buried at the site [78]. The same treatment was given to individuals of the same time period found in surface burials at Taifi (FURS10_Id1) and Kiporu (FURS13_Id1) who show variable levels of affinities with both regions of the Pacific.

Common mortuary treatments and association in the same burial context suggest that Eastern-affiliated individuals were integrated within the local social system by c. 400 BP on Efate and c. 300 BP on Futuna. This result undermines an old hypothesis, based on early ethnographic writtings, under which migrants from Polynesian islands mainly evolved at the margin of the indigenous groups with limited contacts with them [79]. For instance, an oral tradition of the Polynesian Outlier of Rennell (Solomon Islands) mentions that a land was given on Bellona Island to a Polynesian group with the authorization to cultivate it [80]. Our results rather support the alternative hypothesis suggesting that Polynesian individuals were of importance and well-integrated in Vanuatu local societies, favouring opportunities for biological admixture and cultural entanglement.

### Role of Polynesian women in the formation of last millennium Vanuatu societies

In Eretok, as well as in Futuna, the Eastern-Pacific-affiliated individuals are all females (2; 22; 29 and FURS1). All other individuals displaying various levels of Western-Pacific affinities are of both sexes. At Eretok, two of the Eastern-Pacific-affiliated females (2 and 22) are buried each with a man, forming couples (Fig 5). This mortuary arrangement may indicate the reproduction of a situation that existed during their lifetimes and suggests a matrimonial relation between the individuals. Matrimonial exchanges are good examples of a mechanism which allows integration of newcomers within a hosting community as indicated by an oral tradition collected in the Outlier of West Uvea (in the Loyalty Islands), an island lying about 400 km south of West Futuna [81]. For instance, in western Melanesia, the matrilocal Austronesian villages of We-hali (Timor Leste) show that women marry Papuan men, but their offspring adopts the maternal cultural system and are unaware of the language and customs of the paternal lineage [82]. This example highlights the importance of cultural inheritance passed down from migrant-parents to their descendants, which can be suppressed or adopted.

In Central Vanuatu, Chief Roi Mata is said to have initiated a significant social transformation in the structure of the pre-existing societies of the North-West part of Efate by imposing a new socio-political system that favoured the matrilineal transmission of the totem (*naflak* or the emblem of plants or animals) and communal ownership^,^ a system still existing today across Efate. Missionary Peter Milne stated about the naflak: “A man can marry whoever he wants as long as the woman does not belong to his totem, nor is she related to him by blood, no matter how distant” (in [83]). Distinctive elements of Polynesian culture and language could have been spread down the generations thanks to the presence of Polynesian mothers. In Central Vanuatu, the influence of women with a Polynesian culture in a society which values matrilineality likely played a significant role in the formation of cross-cultural communities.

The sland of Futuna was not influenced in a similar fashion by a social change promoting a matriclan-centred society, although a pattern of female exogamy exists between islands of South Vanuatu [18, 22]. Music and songs from Futuna evoke the blessings and heartaches of interisland marriages or point to the departure of local brides towards Polynesia. Several oral histories [18],emphasise the arrival of foreign women (either from the neighbouring islands of Southern Vanuatu or more distant locations) and incorporation in the local society through marriages. We can for example cite the mythical history mentioning that ‘toga’ (meaning foreigner or Tonga) women often fly to Sinou, on the northern coast ofthe island, and one of them was forced to marry Mahjijiki, a mythical figure from the island [18].

Remarkably, all individuals identified as phenotypically Eastern-Pacific-affiliated are females, suggesting that some elements of Polynesian culture were introduced through matrimonial exchanges resulting from ongoing relationships between local populations and populations from the East-Pacific. It is possible that the integration of a few migrants introduced a limited proportion of “Polynesian” biological components within the local community, which may have subsequently reduced over generations in the absence of renewed contacts. This could be one of the explanations for the low visibility of “Polynesian” morphological signals recorded so far in morphometrical studies of ancient and modern populations of Vanuatu, despite the presence of several Outliers in the archipelago.

We suggest that the practice of exogamy with Polynesian individuals was limited in scope and has not trigerred to a major biological shift in the history of the whole Vanuatu population for at least two reasons. First, several genetic studies [1, 2, 41] have demonstrated that the sex-biased admixture between the ancestors of present-day population of Vanuatu is not explained by recent migrations from Polynesia but by an earlier admixture (around 1700–2300 ya [41]). Secondly, the genetic study on 287 couples from Vanuatu has shown that the majority of individuals currently have a partner from the same island as themselves and that spouses tend to share similar genetic ancestry, even if the study tended to support that endogamy is not the norm for Ni-Vanuatu communities [41].

Interestingly, our results echo those of an inter-island mobility study based on an isotopic analysis of individuals from the cemetery of Namu (Taumako, Solomon Islands) [45] suggesting patrilocality and female exogamy. Our small sample and the diversity of oral histories telling of the arrival of individuals of both sexes [81] originating from Polynesia, prevent us from assuming that Polynesian-influenced regions of Vanuatu were strictly patrilocal and that Polynesian migration were sex biased. However, our results allow us to stress the importance of inter-regional exogamy practices for the integration of foreign influences and languages within a community.

## Conclusion

Our results, although obtained from a relatively small sample, demonstrate the effective presence in Central and Southern Vanuatu, during the last 1000 years, of individual whose cranial shape strongly relates to that of modern Polynesian individuals. These findings potentially have direct implications for interpretating cultural changes observed in the region such as the development of monumental structures and the intensification of agricultural systems [14, 84, 85], although other forms of local interactions may also have been involved [15, 22], as well as the development of local identities and social complexity [76]. It is likely that any transformations in Vanuatu’s languages, societies, belief systems, or material culture originating in Polynesia were not solely acquired through indirect interactions, such as exchanges of Polynesian goods and ideas [77, 86, 87].They would have also involved social and biological relationships between people from the East and the populations already settled in Vanuatu. It would be interesting to analyze, on a finer scale of study, the Polynesian micro-regional source of these individuals, by comparing them with morphologically differentiated Polynesian populations [88]. A strontium isotope analysis might also help to clarify the geographical origin of these individuals.

There were likely numerous reasons to leave a homeland situated in Polynesia, includingpolitical disruptions that led to the flight of refugees from struggles for supremacy among powerful chiefdoms in Samoa and Tonga [89, 90], or ecological disasters such as aridity and loss of vital resources. Our study highlights another motivation for voyaging, that of looking for marriage partners as a way of expanding kinship networks. Chiefly conflicts over territory or titles drove Pacific Islander social dynamics that included long-distance voyaging and interactions. Equally, islanders may have sought new islands for the sake of extending family ties across their “sea of islands” [91]. Participating in a process of globalization through long-distance movements, these dispersals may be seen as indicative of a period of increased human interactions in the Western Pacific within the last millennium.

## Supporting information

S1 Appendix Geographical distribution of the modern male and female adult individuals used in this study (n = 232) Western-Pacific: Australia (n = 20), Papua New Guinea (PNG, n = 23), Solomon Islands (n = 7), Vanuatu (n = 23), New-Caledonia (n = 21) and Loyalty Islands (n = 26) (n tot. **= 120).** Eastern-Pacific: Easter Island (n = 22), Marquesas (n = 30), Wallis (East Uvea) (n = 9), Tuamotu Archipelago (n = 22) and Tahiti (n = 27) (n tot = 112). The collections are housed in the Collection d’Anthropologie of the Muséum National d’Histoire Naturelle at the Musée de l’Homme (Paris, France).
(TIF)

S2 Appendix Between-group PCA of 232 modern individuals from 11 populations in Oceania, calculated from the PCA scores of the PCA of 12 size-corrected (LSRs) measurements: Ellipses at 85% for each population from Australo-Melanesia (blue/green) and Polynesia (orange/yellow). NC = New Caledonia; PNG = Papua New Guinea.
(TIF)

S3 Appendix Projection of 12 size-corrected measurements (LSRs) on PC1-PC2, PCA of 232 modern individuals from 2 geographical groups (Polynesia and Australo-Melanesia). (TIF)

S1 Table List of modern male and female adult individuals analyzed in this study with specific origin. (DOCX)

S2 Table 12 Cranial measurements selected for this study (“M” refers to Martin and Sallerdefinitions). (TIF)

S3 Table Summary of cranial measurements (in mm) for each modern population analyzed in the study according to the selected variables. (DOCX)

S4 Table Cranial measurements (in mm) for each archaeological individuals according to the selected variables. (DOCX)

S5 Table Morphological affinities of the archaeological individuals from Eretok and West Futuna: Percents of the predicted probabilities of posterior assignations to the 2 geographical areas: Eastern-Pacific and Western-Pacific. (DOCX)
