# Supplementary figures and images for: “Feeling at home in Vanuatu”: Integration of newcomers from the East during the last millennium
Source: PLoS One. 2024 Jan 31;19(1):e0290465. doi: 10.1371/journal.pone.0290465 (PMC10830024; doi:10.1371/journal.pone.0290465)

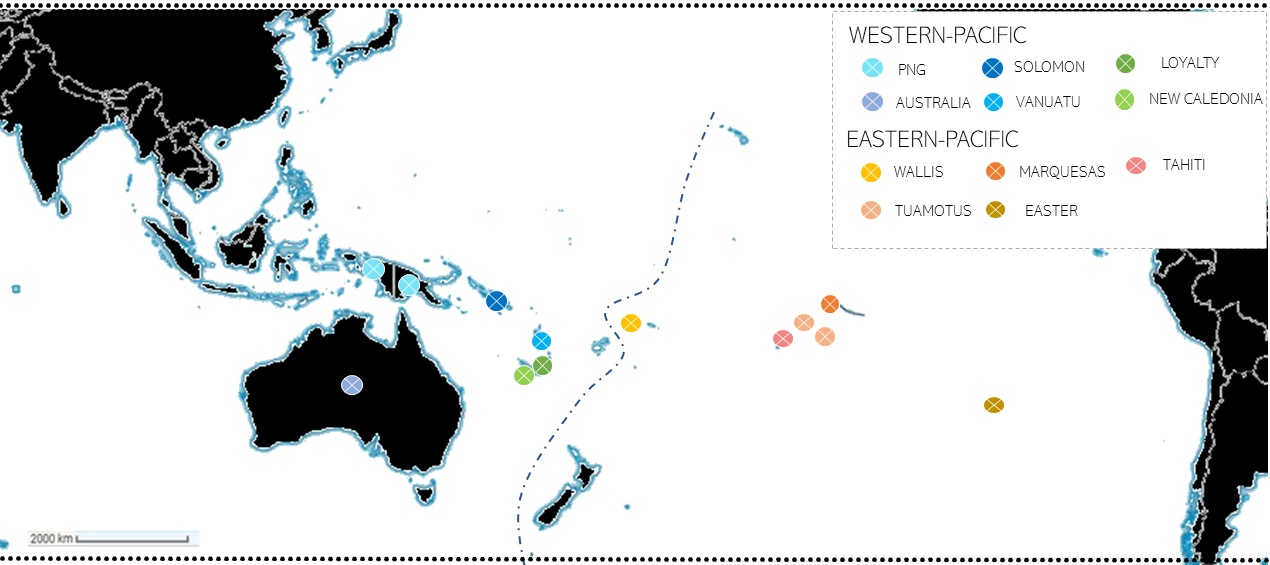

Supplement: S1 Appendix — = 120). Eastern-Pacific: Easter Island (n = 22), Marquesas (n = 30), Wallis (East Uvea) (n = 9), Tuamotu Archipelago (n = 22) and Tahiti (n = 27) (n tot = 112). The collections are housed in the Collection d’Anthropologie of the Muséum National d’Histoire Naturelle at the Musée de l’Homme (Paris, France). (TIF) [file pone.0290465.s001.tif]

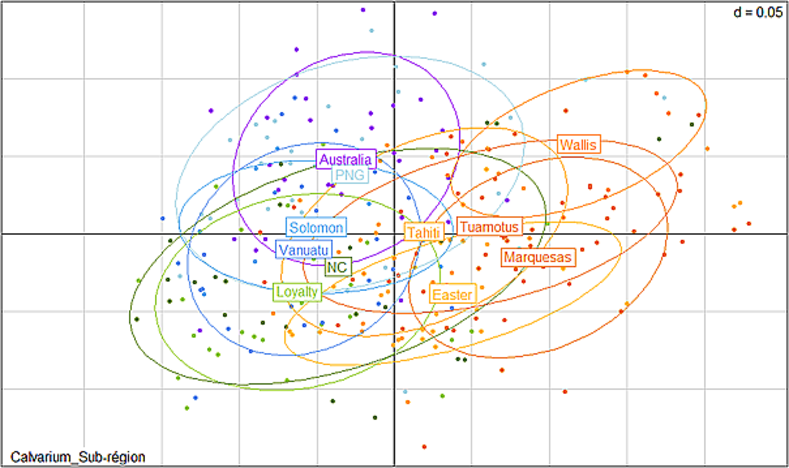

Supplement: S2 Appendix — NC = New Caledonia; PNG = Papua New Guinea. (TIF) [file pone.0290465.s002.tif]

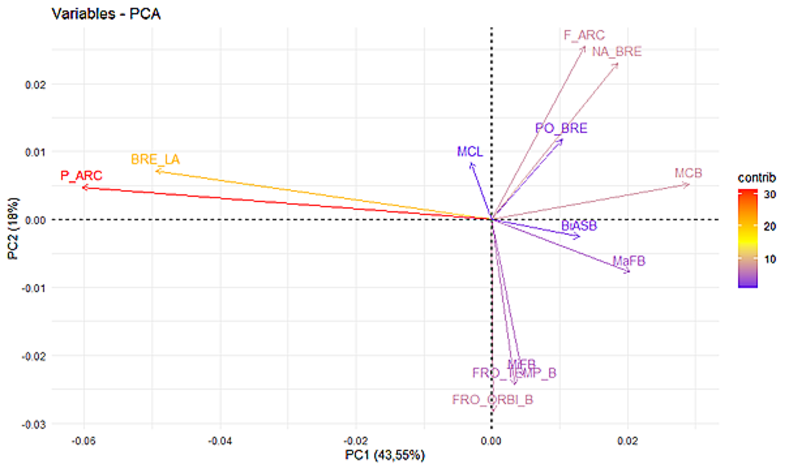

Supplement: S3 Appendix — (TIF) [file pone.0290465.s003.tif]

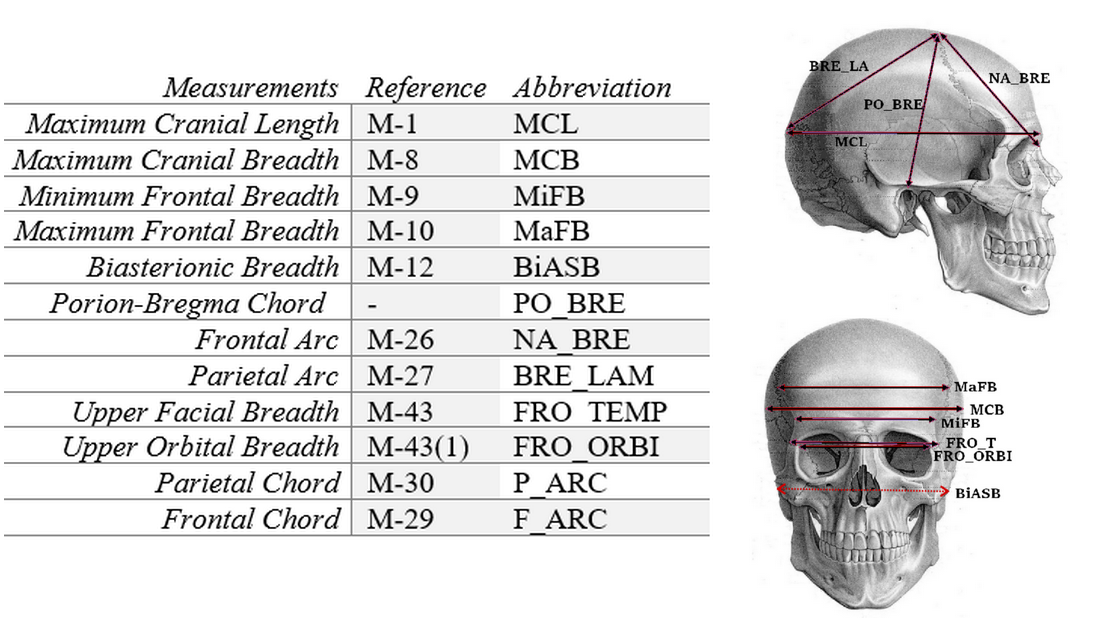

Supplement: S2 Table — (TIF) [file pone.0290465.s005.tif]
