# Supplementary material for: “Feeling at home in Vanuatu”: Integration of newcomers from the East during the last millennium
Source: PLoS One. 2024 Jan 31;19(1):e0290465. doi: 10.1371/journal.pone.0290465 (PMC10830024; doi:10.1371/journal.pone.0290465)
Supplement: S1 Table — (DOCX) [file pone.0290465.s004.docx]

| Region/Area | Number of individuals | Locality |
| --- | --- | --- |
| ***Western-Pacific*** |  |  |
| Australia | 10 | Raffles Bay, King George River, New South Wales, Moreton Bay |
| Papua New Guinea | 28 | Cenderawasih Bay, Triton Bay (Teluk Region), Delta Purari or Purari River, Teba |
| Solomon Archipelago | 8 | Isabel Island, Malaita Island |
| Vanuatu Archipelago | 25 | Malekula Island, Efate Island |
| New Caledonia | 24 | Canala, Boulouparis, La Foa, Pouébo, Koné, Thio, Touho, Yaté, Païta, Mont-Dore, Hienghène, Ponérihouen |
| Loyalty Archipelago | 25 | Mare Island, Lifou Island |
| ***Eastern-Pacific*** |  |  |
| Wallis and Futuna | 8 | Wallis Island (can be called as Uvea), Futuna Island |
| Society Archipelago | 28 | Tahiti Island, Raiatea Island |
| Tuamotus Archipelago | 25 | Anaa island, Kaukura island, Rangiroa, Makatea island (associated to Rangiroa Island) |
| Marquesas Archipelago | 27 | Tahuata Island, Fatu Hiva Island, Nuku Hiva Island |
| Easter Island/ Rapa Nui | 24 | Hanga Ho'onu or Hanga Hoonu, Vaihu or Ahu Hanga Te'e |
