# Supplementary material for: “Feeling at home in Vanuatu”: Integration of newcomers from the East during the last millennium
Source: PLoS One. 2024 Jan 31;19(1):e0290465. doi: 10.1371/journal.pone.0290465 (PMC10830024; doi:10.1371/journal.pone.0290465)
Supplement: S3 Table — (DOCX) [file pone.0290465.s006.docx]

| Specimens |  | MCL | MCB | MiFB | MaFB | BiASB | PO_BRE | BRE_LA | FRO_TEMP_B | FRO_ORBI_B | F_ARC | P_ARC | NA_BRE |
| --- | --- | --- | --- | --- | --- | --- | --- | --- | --- | --- | --- | --- | --- |
| Vanuatu | Mean | 184,226087 | 132,973913 | 95,126087 | 104,669565 | 105,326087 | 127,713043 | 116,659091 | 104,56087 | 97,6304348 | 124,652174 | 131,318182 | 109,604348 |
|  | Min | 171 | 123 | 88,5 | 93 | 95 | 121 | 110 | 97,9 | 91 | 110 | 126 | 98 |
|  | Max | 200 | 144 | 104 | 112 | 114 | 135 | 128 | 112,5 | 104 | 143 | 142 | 120 |
| Loyalty | Mean | 186,508333 | 129,083333 | 92,4166667 | 99,5666667 | 105,0375 | 127,895833 | 117,020833 | 104,708333 | 98,3666667 | 125,541667 | 131,104167 | 112,004167 |
|  | Min | 171 | 124 | 81,5 | 90 | 95 | 117 | 103,5 | 98 | 91 | 117 | 112 | 101 |
|  | Max | 198,5 | 135 | 102 | 107,5 | 113 | 134 | 127 | 113 | 107 | 134 | 146 | 127 |
| New Caledonia | Mean | 186,021739 | 132,63913 | 93,1956522 | 103,456522 | 105,23913 | 130,386957 | 118,621739 | 105,269565 | 98,2695652 | 126,363636 | 134,065217 | 111,93913 |
|  | Min | 172 | 123 | 86,5 | 94,5 | 93,5 | 121 | 112 | 100,9 | 90 | 114 | 125 | 103 |
|  | Max | 202 | 146,5 | 101,5 | 115 | 113 | 141 | 134,5 | 112 | 107 | 137 | 155 | 125,5 |
| Marquesas | Mean | 187,496667 | 143,71 | 93,5533333 | 107,516667 | 109,127586 | 131,01 | 109,243333 | 104,686667 | 97,0833333 | 128,716667 | 120,833333 | 113,27931 |
|  | Min | 174 | 130 | 84 | 102 | 98 | 121 | 95,5 | 92,5 | 88 | 118 | 105 | 102 |
|  | Max | 198 | 154 | 102 | 114 | 117 | 145 | 120 | 112 | 104 | 141 | 133 | 121,5 |
| Papua New Guinea | Mean | 181,604348 | 131,304348 | 96,0695652 | 107,386957 | 106,230435 | 125,891304 | 116,386957 | 106,343478 | 99,2 | 124,413043 | 130,23913 | 115,021739 |
|  | Min | 165,5 | 123 | 87 | 99 | 99,8 | 120,5 | 104,2 | 99 | 92 | 110 | 116 | 106 |
|  | Max | 195 | 141 | 103,5 | 122,5 | 117,5 | 133 | 126,5 | 114 | 105,5 | 137 | 142 | 129 |
| Tuamotus | Mean | 178,543478 | 138,195652 | 93,0217391 | 106,373913 | 106,008696 | 128,695652 | 107,004348 | 102,621739 | 94,3043478 | 127,304348 | 117,73913 | 113,804348 |
|  | Min | 165,5 | 131 | 87,5 | 95 | 99,5 | 121,5 | 95 | 95 | 88 | 116 | 100 | 103 |
|  | Max | 190 | 147,5 | 102 | 117 | 113 | 136 | 116 | 109,5 | 101 | 136 | 130 | 122 |
| Solomon | Mean | 177,714286 | 126,428571 | 93,5 | 101,585714 | 105,214286 | 125,571429 | 113,1 | 103,214286 | 97,2857143 | 122,857143 | 129,142857 | 109,857143 |
|  | Min | 168 | 123 | 88 | 97 | 97,5 | 118 | 107 | 97 | 93,5 | 117 | 119 | 102,5 |
|  | Max | 182 | 130 | 99 | 107 | 109 | 130 | 120 | 110 | 100 | 128 | 136 | 115,5 |
| Wallis | Mean | 177,785714 | 142,033333 | 94,4285714 | 112,642857 | 107,071429 | 131,528571 | 104,814286 | 106,4 | 98,5 | 127,428571 | 118 | 112,67 |
|  | Min | 168,5 | 134,2 | 89 | 106,5 | 97,5 | 126 | 95 | 99,3 | 93 | 122 | 104 | 103 |
|  | Max | 189 | 151 | 100,5 | 119 | 118 | 136 | 116,2 | 114 | 105 | 133 | 130 | 120 |
| Easter Island | Mean | 188,879167 | 135,734783 | 92,8083333 | 105,817391 | 105,408333 | 134,166667 | 111,020833 | 104,029167 | 96,3541667 | 129,75 | 122,125 | 109,175 |
|  | Min | 174,1 | 125 | 86,5 | 93 | 94 | 125 | 90 | 97 | 88 | 122 | 94 | 98,2 |
|  | Max | 207,5 | 145 | 102,8 | 111 | 113,5 | 142 | 120 | 113 | 103,5 | 140 | 135 | 120,5 |
| Tahiti | Mean | 182,4 | 138,75 | 94,3333333 | 107,546667 | 106,076667 | 130,633333 | 113,276667 | 105,32 | 97,65 | 128,733333 | 127,333333 | 111,213333 |
|  | Min | 168 | 128 | 87 | 101 | 98 | 121 | 97,5 | 96,1 | 88,5 | 114 | 104 | 98,5 |
|  | Max | 194,4 | 150 | 104,5 | 117,5 | 115 | 142 | 124 | 115 | 105 | 146 | 138 | 121,1 |
| Australia | Mean | 181,055 | 130,125 | 95,56 | 103,685 | 104,915 | 122 | 112,835 | 109,01 | 102,075 | 126,15 | 125,45 | 109,85 |
|  | Min | 164,5 | 121 | 88,5 | 94,5 | 93 | 110 | 102,9 | 101,5 | 95,5 | 114 | 116 | 104,5 |
|  | Max | 201 | 137 | 103 | 110 | 113 | 131 | 125,5 | 119 | 110 | 138 | 142 | 116,1 |
