# Supplementary material for: “Feeling at home in Vanuatu”: Integration of newcomers from the East during the last millennium
Source: PLoS One. 2024 Jan 31;19(1):e0290465. doi: 10.1371/journal.pone.0290465 (PMC10830024; doi:10.1371/journal.pone.0290465)
Supplement: S4 Table — (DOCX) [file pone.0290465.s007.docx]

| Individuals | MCL | MCB | MiFB | MaFB | BiASB | PO_BRE | NA_BRE | BRE_LA | FRO_TEMP_B | FRO_ORBI_B | F_ARC | P_ARC |
| --- | --- | --- | --- | --- | --- | --- | --- | --- | --- | --- | --- | --- |
| 25795 | 182 | 132 | 96 | 107 | 107 | 131 | 119 | 115 | 105 | 97 | 132 | 130 |
| 25792 | 181 | 135 | 86 | 104 | 102 | 129 | 115 | 119 | 99 | 94,5 | 124 | 133 |
| 25796 | 183,5 | 126 | 93,5 | 103 | 106 | 129 | 111 | 118 | 102 | 99 | 127 | 130 |
| 25793 | 183 | 140 | 99 | 114 | 102 | 135 | 116 | 120 | 106 | 98 | 128 | 161 |
| 25798 | 182 | 137 | 90 | 110 | 103 | 124 | 114 | 106 | 98 | 90 | 128 | 115 |
| 25797 | 181 | 130 | 99 | 104 | 110 | 134 | 112 | 120 | 112 | 104 | 124 | 128 |
| 25791 | 182 | 129 | 96 | 111 | 110 | 127 | 117 | 119,5 | 106 | 99 | 131 | 125 |
| 25800 | 189 | 139 | 91 | 103 | 99 | 136 | 115 | 120 | 103 | 96 | 125 | 131 |
| 25799 | 188 | 127 | 97 | 109 | 112 | 131 | 109 | 126,5 | 104,5 | 100 | 120 | 140 |
| 25794 | 187 | 133 | 95 | 102 | 106 | 129 | 114,5 | 122 | 101 | 103 | 126 | 135 |
| FURS1 | 186 | 133,5 | 93 | 103 | 100 | 128 | 113 | 111 | 101 | 92 | 128 | 120 |
| FURS13 | 168 | 134 | 89 | 92 | 105 | 118 | 103,5 | 107 | 97,2 | 88 | 117 | 120 |
| FURS10_Id1 | 174,5 | 133,5 | 90,5 | 101,5 | 104,5 | 123 | 109,5 | 113 | 102,5 | 96 | 122 | 128 |
