# Supplementary material for: “Feeling at home in Vanuatu”: Integration of newcomers from the East during the last millennium
Source: PLoS One. 2024 Jan 31;19(1):e0290465. doi: 10.1371/journal.pone.0290465 (PMC10830024; doi:10.1371/journal.pone.0290465)
Supplement: S5 Table — (DOCX) [file pone.0290465.s008.docx]

| Archaeological Individual | % of assignation to morphological trends | |
| --- | --- | --- |
|  | EASTERN-PACIFIC | WESTERN-PACIFIC |
| 12 | 24 | 76 |
| 2 | 90 | 10 |
| 15 | 2 | 98 |
| 6 | 98 | 2 |
| 2bis | 17 | 83 |
| 22 | 100 | 0 |
| 19 | 1 | 99 |
| 1 | 7 | 93 |
| 29 | 97 | 3 |
| 26 | 0 | 100 |
| Unknown | 5 | 95 |
| FURS1_Id1 | 93 | 7 |
| FURS13_Id1 | 48 | 52 |
| FURS10_Id1 | 22 | 78 |
